# CastNet: a systems-level sequence evolution simulator

**DOI:** 10.1186/s12859-023-05366-1

**Published:** 2023-06-12

**Authors:** Carlos J. Rivera-Rivera, Djordje Grbic

**Affiliations:** 1 University of Bristol, Life Sciences Building, 24 Tyndall Avenue, Bristol, BS8 1TG UK; 2 IT-University of Copenhagen, Rued Langgaards Vej 7, 2300 Copenhagen, Denmark

**Keywords:** Evolution simulation, Evo-devo, Complex systems, Gene regulatory networks, Evolutionary systems, Coevolutionary webs, Coevolution

## Abstract

**Background:**

Simulating DNA evolution has been done through coevolution-agnostic probabilistic frameworks for the past 3 decades. The most common implementation is by using the converse of the probabilistic approach used to infer phylogenies which, in the simplest form, simulates a single sequence at a time. However, biological systems are multi-genic, and gene products can affect each other’s evolutionary paths through coevolution. These crucial evolutionary dynamics still remain to be simulated, and we believe that modelling them can lead to profound insights for comparative genomics.

**Results:**

Here we present CastNet, a genome evolution simulator that assumes each genome is a collection of genes with constantly evolving regulatory interactions in between them. The regulatory interactions produce a phenotype in the form of gene expression profiles, upon which fitness is calculated. A genetic algorithm is then used to evolve a population of such entities through a user-defined phylogeny. Importantly, the regulatory mutations are a response to sequence mutations, thus making a 1–1 relationship between the rate of evolution of sequences and of regulatory parameters. This is, to our knowledge, the first time the evolution of sequences and regulation have been explicitly linked in a simulation, despite there being a multitude of sequence evolution simulators, and a handful of models to simulate Gene Regulatory Network (GRN) evolution. In our test runs, we see a coevolutionary signal among genes that are active in the GRN, and neutral evolution in genes that are not included in the network, showing that selective pressures imposed on the regulatory output of the genes are reflected in their sequences.

**Conclusion:**

We believe that CastNet represents a substantial step for developing new tools to study genome evolution, and more broadly, coevolutionary webs and complex evolving systems. This simulator also provides a new framework to study molecular evolution where sequence coevolution has a leading role.

**Supplementary Information:**

The online version contains supplementary material available at 10.1186/s12859-023-05366-1.

## Background

DNA and amino acid sequence evolution have traditionally been simulated through probabilistic models that infer substitutions on a single sequence in a branch-independent manner [1–5]. These methods do not allow for gene coevolution by principle, since they simulate the evolution of a single sequence, one branch at a time [6, 7], impeding by design the simulation of coevolving sequences. Biological evolution, however, has a diversity of examples in which sequence coevolution within and across lineages is evident and often pervasive (e.g. [8–10]).

The evolution of networks of interacting genes, or gene regulatory networks (GRNs) has been studied with simulations since the landmark models of Wagner [11, 12]. These studies, and related ones that followed (e.g. [13–16]) focus on the structural properties of GRNs that emerge due to the evolutionary process. One of these studies identified that robustness to mutation can emerge spontaneously through evolution, via the modification the GRN topology, thus ensuring consistent developmental results from networks that were slightly dissimilar [13]. Many of these studies find that structural properties of the GRNs affect a system’s access to phenotypic spaces, or its evolvability, a crucial component of evolving systems we should understand in an environment that is changing at an ever-faster pace. The insights into evolutionary dynamics these studies achieved are ground-breaking and, in our view, still warrant additional study.

The studies of sequence and GRN evolution have remained separate so far. The most obvious reasoning for this is that the evolution of regulation is expected to influence regulatory sequences, rather than protein-coding sequences. However, biological examples of sequence coevolution observed directly on protein-coding sequences led us to propose a simulator in which GRN evolution and sequence evolution are explicitly linked. This simulator enables exploration into the extent to which the coevolutionary links among gene products constrain or channel sequence evolution. Such a system also allows for a simulation-driven test of the analytical proofs by González-Forero [17], and can help to determine whether developmental information can be extracted solely from sequence evolution.

## Implementation

Here we present CastNet, a highly customizable software for simulating the evolution of virtual organisms composed of genomes and proteomes, where genes are functionally linked.

### Virtual organism structure

CastNet creates a genome of a user-defined size in nucleotides, *n* × *l* (*n* = number of genes, *l* = sequence length in base pairs). The simulated genome is built by randomly sampling codons under a uniform distribution. CastNet then creates a regulatory matrix ***R*** of size *n* × *n*, which encodes the regulatory effects each gene of the system has on all other genes, and the regulatory effect that all genes have on each gene. The values in this matrix are sampled from a uniform distribution within a user-defined range that ideally includes negative (downregulation/repression) as well as positive (upregulation/activation) values, and then a mask is used to turn a user-defined proportion of cells to 0, to enforce sparseness. There are two additional gene-specific regulatory parameters: (1) a threshold $\theta$ indicating the minimum activating signal that needs to be received by each gene in order for it to be expressed (from Espinosa-Soto 2008 [16]), and (2) A gene-specific decay rate $\lambda$ determining how quickly each gene’s amount decreases with time under an exponential decay model.

### Simulating development

With the regulatory parameters ${\varvec{R}}$, ${\varvec{\lambda}} = \user2{ }\left[ {\lambda_{G1,} \lambda_{G2} , \ldots ,\lambda_{Gn} } \right]$, and ${\varvec{\theta}} = \left[ {\theta_{G1} ,\theta_{G2} , \ldots ,\theta_{Gn} } \right]$, CastNet simulates the development of a system through a time-discrete Markov Chain by calculating the gene quantities vector ${\varvec{q}}_{t}$ at each step $t$, following:

1 $${\varvec{q}}_{{\varvec{t}}} = \left( {{\varvec{ReLU}}_{{\uptheta }} \left( {{\mathbf{R}} \cdot {\varvec{q}}_{{{\varvec{t}} - 1}} } \right) + {\varvec{q}}_{{{\varvec{t}} - 1}} e^{{ - {\uplambda }}} } \right)*{\varvec{c}}$$

where ${\varvec{q}}_{t} = \left[ {q_{G1} \left( t \right),q_{G2} \left( t \right), \ldots ,q_{Gn} \left( t \right)} \right]$ is a vector of size $n$ with the quantity $q_{Gi} \left( t \right)$ of each gene at time $t$ (quantities are in arbitrary units), ${\varvec{q}}_{t - 1} = \left[ {q_{G1} \left( {t - 1} \right),q_{G2} \left( {t - 1} \right), \ldots ,q_{Gn} \left( {t - 1} \right)} \right]$ is the gene quantities in the prior step of the chain, $ReLU_{\theta }$ is a rectifier function that rectifies to 0 any value below each gene’s activation threshold $\theta_{Gi}$, and ${\varvec{c}} = \left[ {{\varvec{c}}_{G1} ,{\varvec{c}}_{G2} , \ldots ,{\varvec{c}}_{Gn} } \right]$ is a vector of size $n$ that has a zero if the gene has a stop codon, and one otherwise. ${\varvec{c}}$ multiplies by 0 the quantities of any gene which shows a nonsense mutation (i.e. with a stop codon mid-sequence), and by one that of genes with an intact open reading frame. This Markov chain is run for a user-defined number of developmental steps $m$, using an arbitrary seed quantity of 1 for the first gene in the list, and 0 for all other genes. This recursive operation results in an expression matrix ${\varvec{E}}$ of size $n$ × $m$, which can be interpreted as gene expression profiles for all genes in the system (Algorithm 1). This simulation of development assumes that all genes are monoexonic and have single isoform, and that regulation is happening exclusively at the level of transcription (similar to Wagner’s model in [11]).

**Figure:**
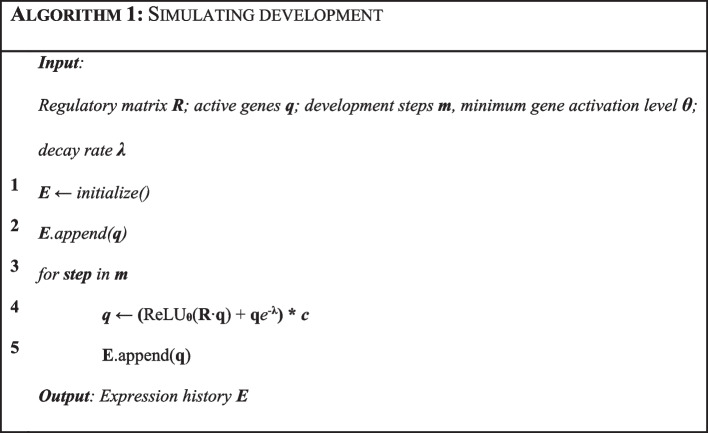

A standard genetic algorithm (GA) [18] is used to simulate evolution in this system (Algorithm 2). Broadly, the mutation of an organism in each generation occurs in the following steps:

1. Mutate the genome sequence based on a user-defined ‘probability of a point mutation per base, per generation’ rate $\mu$.
2. Assign each mutation to the gene in which it happened, and whether they are synonymous, non-synonymous, or nonsense mutations.
3. For each mutation, mutate any regulatory value from the corresponding gene, reflecting their synonymous or non-synonymous nature. Non-synonymous mutations will occur in links which are known to be active (e.g., a gene that is expressed in the parental expression matrix ${\varvec{E}}$), whereas synonymous mutations would occur in links which are known to be inactive (e.g., parental expression matrix ${\varvec{E}}$ shows no activation of that specific link). Synonymous regulatory mutations are intended to allow for ‘under the hood’ or hidden regulatory changes, which do not have a measurable effect in the actual GRN but would allow for larger innovations if the mutated gene is activated.. Whether a mutation occurs on regulatory parameters ${\varvec{\theta}}$ or ${\varvec{\lambda}}$ is determined by the proportion of regulatory values these two vectors represent ($\frac{2}{2 + n}$, where $n$ is the number of genes).

The following assumptions are in place in the mutational process: 1. Point mutations are equally likely across the genome (i.e. no site rate heterogeneity), and each mutation will result in any of the other three nucleotides with equal probability (i.e. follows a JC model), 2. The *location* of a mutation in the sequence is independent from the regulatory mutation, 3. The reproductive system is clonal, haploid, and does not have recombination, 4. Insertions/deletions, and gene/genome duplications do not occur, 5. Regulatory, evolutionary and fitness parameters are branch-specific unless otherwise enforced by the user.

The phenotype upon which fitness is measured is the output of the regulatory parameters (i.e., the gene expression profiles). Four metrics are used to evaluate the fitness of each gene expression profile: gene quantities must not increase exponentially; the more genes activated in the system, the fitter the organism; gene expression should be as stable as possible; and the system must have reached maturity (indicated by an arbitrary gene having passed a user-defined threshold in quantity, and being expressed in the final developmental step). Specific details on how these metrics are calculated are in Additional file 1, and in the manual at the CastNet homepage.

CastNet then allows populations of such systems to evolve through user-defined branches, and numbers of generations, and in the end, will have multiple sequences simulated for each GRN, following the selective pressures imposed on the gene expression profile. CastNet simulates the evolution of multi-genic systems branch by branch, and the way the outputs are organized makes changing evolutionary parameters mid-branch relatively easy. This feature permits the simulation of branch-specific (or generation-specific within one branch) changes in rates of evolution, population size, and selective strategies.

**Figure:**
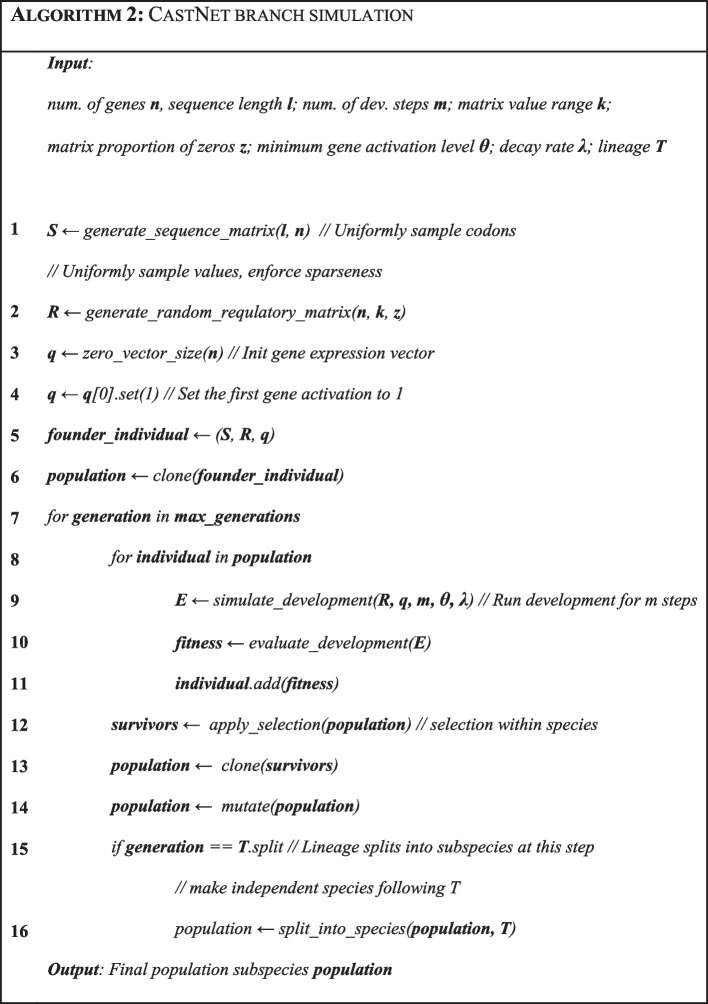

## Results

**Fig. 1:**
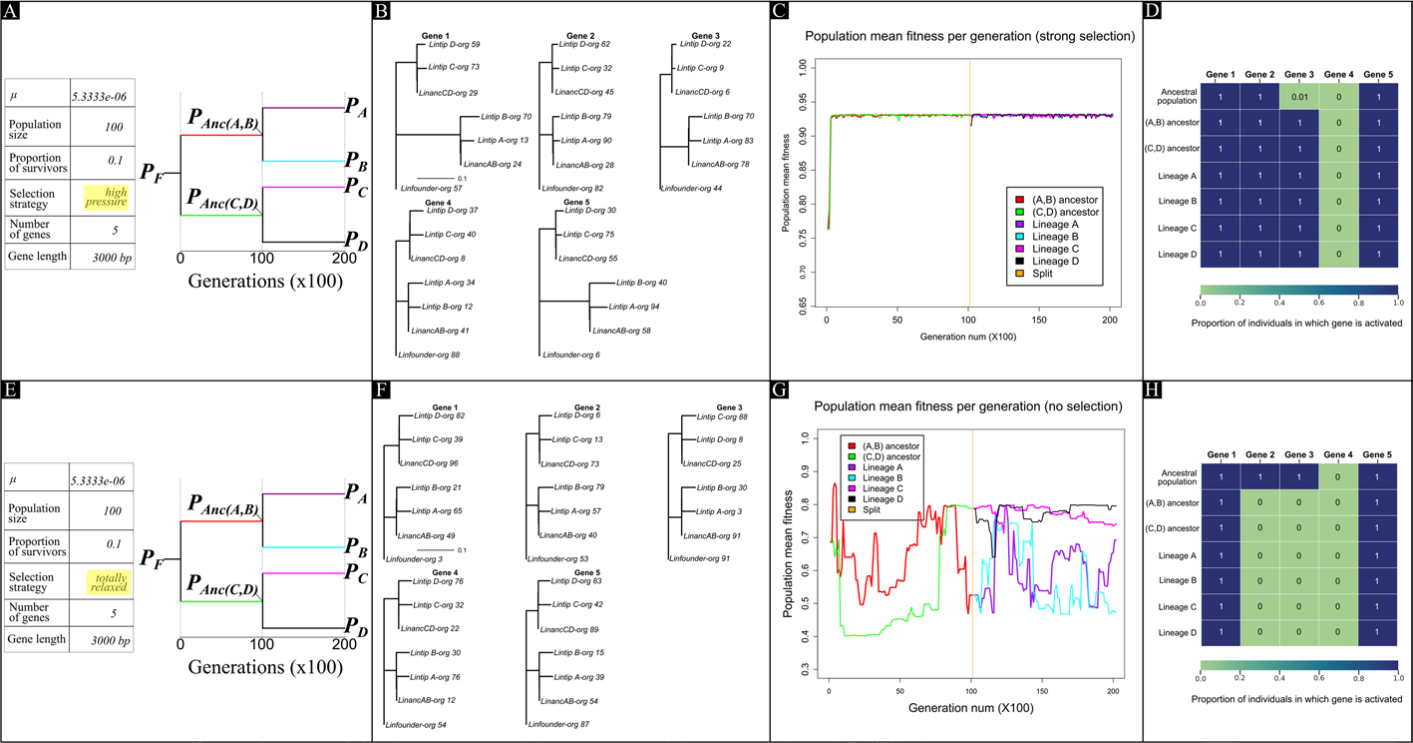
Experimental data from Runs 1 and 2 of the CastNet proof-of-concept. **A**–**D** shows results for Run 1 (stringent selection), and **E**–**H** for Run 2 (no selection). **A** and **E** show the target phylogenies, **B** and **F** the gene trees, **C** and **G** the mean population fitness progression (sampled every 100 generations, for 20,000 generations), and **D** and **H** in what proportion of the population each gene was expressed

We ran a proof con concept (POC) of CastNet with two simulated test runs of four lineages, five genes of 3000 bp each, and 20,000 generations, using a fully symmetrical tree (Fig. 1A and E), a selection proportion of *p* = *0.1*, but with two different selection strategies: ‘high pressure’ (only fittest $p$ survive—Run 1), and ‘totally relaxed’ ($p$ random organisms survive—Run 2). Our main interest with this proof of concept is to ensure realistic results at three main levels: 1. sequence evolution simulation, 2. GRN and development evolution simulation, and 3. the link between sequence and regulatory mutations. For the mutation rate per base per generation *μ*, we used as a conservative measure, 5.33333e-6 (this represents a suitable starting point for a genome of this size—but see manual for detailed recommended settings for this and all other parameters, as well as benchmarks on how time increases with increasing parameter values). This rate, over the 20,000 generations is expected to produce patristic distances of 0.1066666 subtitutions per site if no selection is present. We cannot provide a fair comparison of these POCs with pre-existing probability-based sequence evolution simulators because functional sequence co-evolution—CastNet’s main feature—is not included in the most widely used simulators.

### Run 1: stringent selection

Sequence evolution was simulated effectively in parallel for all five genes, as all gene trees show the intended topology (Fig. 1B). Interestingly, there are gene-specific evolutionary patterns, although larger replicates are needed to determine how they reflect the evolution of regulatory parameters. Ideally, one would have multiple replicates for statistical power, as well as a reliable metric that compares gene trees within tree space and identifies co-variation of branch lengths. Combined with the (known) variation of the regulatory parameters, one could identify patterns that link regulatory evolution to sequence evolution. In this POC, regulatory parameter evolution was also successfully simulated, as it is reflected in the mean fitness traces with strong positive selection in the first few thousand generations, and a stabilization in a local optimum for the remainder of the run on all branches (Fig. 1C). Finally, gene participation is strongly selected for, as in the founder population, Gene 3 is only activated in 1% of the population, whereas in all subsequent lineages this number is 100% (Fig. 1D). The fact that Gene 4 is never activated is also evident in the number of stop codons each lineage’s sequence has (Lineage A: 13, Lineage B: 12, Lineage C: 12, Lineage D: 15), showing that sequences of genes not involved in the normal operation of gene expression will accumulate stop codons, as would be expected from a silenced gene. Thus, this simulation run confirms that CastNet is effective at all three levels initially sought: simulating sequence evolution, simulating the evolution of regulation, and passing selective signals from the regulatory network onto the genomic sequence.

### Run 2: no selection

Without selection, gene trees still follow the correct phylogeny, but no longer show gene-specific branch length patterns (Fig. 1F). The generalized branch length pattern confirms the hypothesis stated above that under no selection, the patristic distances of the tip lineages should be close to 0.1. The stochastic way of exploring the fitness landscape when there is no selection is clear on (Fig. 1G), where it progresses in a much less structured way than on Fig. 1D, where selection was present. In addition, we also noticed that selection for gene participation was virtually non-existent in comparison to Run 1, as most genes were inactive throughout most of the tree (Fig. 1H). The only ones still expressed are Genes 1 and 5, which are both needed in order for a system to exist, and will be present regardless of nonsense mutations in their sequences. In this case, Gene 1 indeed has accumulated nonsense mutations (Gene 1 is always expressed because it is the one that kick-starts development), therefore under more realistic conditions this whole population would have potentially gone extinct.

## Conclusion

The sequence simulator presented can effectively simulate the evolution of coding sequences which coevolve due to functional links and have a concrete output as developmental profiles. We envisage that CastNet will serve to address many new and old questions from phylogenetics; for example, to what extent can coevolutionary interactions between genes or systems (regulatory or otherwise) be modelled by measuring sequence covariation through time (given appropriate methods and sample sizes). Additionally, CastNet sets ‘number of generations’ and ‘mutation rate’ as separate parameters, unlike the probabilistic models currently used to infer and ultimately simulate phylogenies, in which branch length is a combination of both values. This feature enables a rigorous exploration of systematic biases like long branch attraction, and how to better manage them, as well as testing for the accuracy of time-tree inference programs, which are methods designed to reliably disentangle time (= number of generations) and mutation rate from observed samples.

## Supplementary Information

**Additional file 1.** Detailed explanation of all fitness criteria, and how they are combined to produce one value.

## Data Availability

The CastNet script as well as the datasets used in these papers and the R and Python scripts used to make the results figures are available on https://github.com/carlosj-rr/CastNet. Project name: CastNet. Project homepage: https://github.com/carlosj-rr/CastNet. Operating system: Platform independent. Programming language: Python 3.9.7. Other requirements: NumPy 1.20.3, Matplotlib 3.5.2, tqdm 4.64.1, scipy 1.9.0, gif 22.5.0. License: GNU GPL v3. Any restriction to use by non-academics: license needed.

## References

[1] Yang Z (2007). PAML 4: phylogenetic analysis by maximum likelihood. Mol Biol Evol.

[2] Rambaut A, Grassly NC (1997). Seq-gen: An application for the monte carlo simulation of dna sequence evolution along phylogenetic trees. Bioinformatics.

[3] Hall BG (2008). Simulating DNA coding sequence evolution with EvolveAGene 3. Mol Biol Evol.

[4] Fletcher W, Yang Z (2009). INDELible: a flexible simulator of biological sequence evolution. Mol Biol Evol.

[5] Ly-Trong N, Naser-Khdour S, Lanfear R, Minh BQ (2021). AliSim: a fast and versatile phylogenetic sequence simulator for the genomic era. Mol Biol Evol.

[6] Yang Z, Rannala B (2012). Molecular phylogenetics: principles and practice. Nat Rev Genet.

[7] Yang Z. Simulating molecular evolution. In: Molecular evolution: a statistical approach. 2014. Oxford University Press (Oxford, UK). p. 418–41.

[8] Yeang CH, Haussler D (2007). Detecting coevolution in and among protein domains. PLoS Comput Biol.

[9] Weber AAT, Abi-Rached L, Galtier N, Bernard A, Montoya-Burgos JI, Chenuil A (2017). Positive selection on sperm ion channels in a brooding brittle star: consequence of life-history traits evolution. Mol Ecol.

[10] Crotty SM, Minh BQ, Bean NG, Holland BR, Tuke J, Jermiin LS (2020). GHOST: recovering historical signal from heterotachously-evolved sequence alignments. Syst Biol.

[11] Wagner A (1994). Evolution of gene networks by gene duplications: a mathematical model and its implications on genome organization. Proc Natl Acad Sci USA.

[12] Wagner A (1996). Does evolutionary plasticity evolve?. Evolution (N Y).

[13] Ciliberti S, Martin OC, Wagner A (2007). Robustness can evolve gradually in complex regulatory gene networks with varying topology. PLoS Comput Biol.

[14] Fierst JL, Phillips PC (2015). Modeling the evolution of complex genetic systems: the gene network family tree. J Exp Zool Part B Mol Dev Evol.

[15] Kittelmann S, Buffry AD, Franke FA, Almudi I, Yoth M, Sabaris G (2018). Gene regulatory network architecture in different developmental contexts influences the genetic basis of morphological evolution. PLoS Genet.

[16] Espinosa-Soto C (2018). On the role of sparseness in the evolution of modularity in gene regulatory networks. PLoS Comput Biol.

[17] González-Forero M (2023). How development affects evolution. Evolution (N Y).

[18] Mitchell M (1998). An introduction to genetic algorithms.

