# Supplementary material for: CastNet: a systems-level sequence evolution simulator
Source: BMC Bioinformatics. 2023 Jun 12;24:247. doi: 10.1186/s12859-023-05366-1 (PMC10259028; doi:10.1186/s12859-023-05366-1)
Supplement: Supplementary file 1 — Additional file 1. Detailed explanation of all fitness criteria, and how they are combined to produce one value. [file 12859_2023_5366_MOESM1_ESM.docx]

**Additional File 1 for:**

**CastNet: a systems-level sequence evolution simulator**

Carlos J. Rivera-Rivera^1^*, Djordje Grbic^2^

^1^ University of Bristol, Life Sciences Building, 24 Tyndall Avenue, BS8 1TG, Bristol, United Kingdom

^2^ IT-University of Copenhagen, Rued Langgaards Vej 7, 2300 Copenhagen, Denmark

*This Supplementary Information File Includes:*

Detailed explanation of all fitness criteria, and how they are combined to produce one value.

**Fitness criteria**

Fitness is defined as the mean of metrics a, b, and c, times metric d.

*a. Non-exponential gene expression*

The sum of all genes’ expression per developmental step should not resemble an exponential curve. This penalises GRNs (Gene Regulatory Networks) with unrealistic amounts of feed-forward loops. The value used is $1-r^{2}$, where $r^{2}$ is the coefficient of determination of a linear regression done on the column-wise sum of gene quantities, transformed into logarithmic space.

*b. Proportion of genes expressed*

The percent of the total genes expressed. Gene expression profiles in which more genes are activated score higher than those in which less are active. The value is just the proportion of the total of genes expressed, and it is meant to correct for sparsely connected GRNs.

*c. Stable expression of genes*

A linear regression is done for each gene’s expression pattern, and the slope is calculated. Each slope is expressed as a ratio of 90, and the average across all genes is the value used. Briefly, a linear model is fitted to the results of each gene’s expression, from which a vector of gene expression slopes, ***m =* [*m_G0_, m_G1_, …, m_Gn_*]**, (where *n* is the total number of genes) is extracted*.* To determine how steep each slope is, we express the angle created by it as a ratio of 90*°*. This way, a gene with a stable expression will approach a value of 0, and a gene with an expression that sharply increases or decreases will approach a value of 1. All the gene-specific ratios to 90*°* are then averaged to get an index for an entire system. The final value used is one minus the average of all genes’ expression.

*d. System maturity*

An arbitrary gene is expected to have been expressed at some point, and to be above a certain user-defined amount in the last developmental step. Representing the ‘maturity’ of a system, or its readiness to reproduce, this metric results in a 1 or a 0, effectively determining whether the system can pass on to the next generation. By design, this is the last gene in the list, but in principle it can be any one gene. This implies that, even when selection is not present based on fitness, there exists a fitness landscape in which Gene 1 and the last gene of the list will be expressed.

We are aware that more metrics can be added, as well as scaling factors that would enable some metrics to become more important than the others, allowing for the exploration of more complex fitness landscapes.
